# Theory and practice of social norms interventions: eight common pitfalls

**DOI:** 10.1186/s12992-018-0398-x

**Published:** 2018-08-17

**Authors:** Beniamino Cislaghi, Lori Heise

**Affiliations:** 1 Department of Global Health and Development, London School of Hygiene and Tropical Medicine, 15-17 Tavistock Place, London, WC1H 9SH UK; 2 Department of Population, Family and Reproductive Health, Johns Hopkins Bloomberg School of Public Health and School of Nursing, 615 N Wolfe Street, Baltimore, MD 21205 USA

**Keywords:** Social norms, NGO, Intervention, Health promotion

## Abstract

**Background:**

Recently, Global Health practitioners, scholars, and donors have expressed increased interest in “changing social norms” as a strategy to promote health and well-being in low and mid-income countries (LMIC). Despite this burgeoning interest, the ability of practitioners to use social norm theory to inform health interventions varies widely.

**Main body:**

Here, we identify eight pitfalls that practitioners must avoid as they plan to integrate a social norms perspective in their interventions, as well as eight learnings. These learnings are: 1) Social norms and attitudes are different; 2) Social norms and attitudes can coincide; 3) Protective norms can offer important resources for achieving effective social improvement in people’s health-related practices; 4) Harmful practices are sustained by a matrix of factors that need to be understood in their interactions; 5) The prevalence of a norm is not necessarily a sign of its strength; 6) Social norms can exert both direct and indirect influence; 7) Publicising the prevalence of a harmful practice can make things worse; 8) People-led social norm change is both the right and the smart thing to do.

**Conclusions:**

As the understanding of how norms evolve in LMIC advances, practitioners will develop greater understanding of what works to help people lead change in harmful norms within their contexts. Awareness of these pitfalls has helped several of them increase the effectiveness of their interventions addressing social norms in the field. We are confident that others will benefit from these reflections as well.

## Background

Practitioners and scholars working for global health have long been interested in understanding how socio-cultural context influences people’s health-related behaviour.

Most of the field’s conceptual models acknowledge both the importance of context in shaping behaviour, and the role of social norms. Brofenbenner’s socio-ecological model, for instance, emphasizes how individual, social, institutional, and macro level factors combine to influence people’s actions [1, 2]. The framework invites users to examine how the micro, meso, and macro-level environments interact to affect human behaviour. It has been used to examine a wide range of health practices “e.g. [3–8]” and more recently adapted specifically to help design social norms interventions [9]. In addition to the ecological model, several other theories and frameworks exist to study how social factors influence people’s health and health-related behaviours [10]. The fields of medical sociology and social epidemiology, for instance, have offered several paradigms of how “social determinants” combine with an individual’s genetic endowment, and social world to make people either healthy or ill [11]. Approaches that analyse social determinants traditionally evoke factors such as income and income distribution, physical environment, employment and job security, education as well as social networks [12].

When these and other behavioural science models have been used to design health-promotion programmes, the relational dimension of social norms has sometimes been lost; rather interventions have focussed instead on improving knowledge and changing attitudes [13–16]. Today, we are witnessing an increased interest in using social norms frameworks to inform health promotion interventions in low and mid-income countries (LMIC) [15, 17–23]. The first “social norms interventions” were originally used to reduce alcohol consumption in a few US universities in the early 1980s by correcting students’ overestimates of how much other students drink [24]. Later, more campuses (mostly in the US, Canada, and the UK) used similar interventions to address tobacco use [25], sexual violence [26, 27], and use of recreational drugs [28].

In the late 90s, several health practitioners working in LMIC began to explore the potential of changing social norms to achieve a wider range of health outcomes, especially around harmful traditional practices. This interest followed the discovery that people in West Africa were abandoning female genital cutting – a non-medically-justified modification of women’s genitalia – in response to interventions that integrated a norms component [29, 30]. Today, many interventions in LMIC aim to change social norms that sustain harmful practices, including, to cite a few examples, child marriage [18, 31, 32], female genital cutting [33, 34] and intimate partner violence [35]. Gelfand and Jackson [36] spoke of this interest as the “emerging science” of social norms, and Miller and Prentice [15] attributed it to a “growing disillusionment with the capacity of factual information and economic inducements to reduce [harmful] behaviour” (p. 340).

Despite this burgeoning interest, the ability of practitioners to use social norm theory to inform health-related interventions varies widely. In practice, we have noticed that when practitioners first apply a social norms frame to behaviour change, they make similar mistakes. To help avoid these early missteps, we outline here, eight common pitfalls that practitioners may encounter in their early efforts to apply social norms theory. We begin my reviewing some of the extensive and multi-disciplinary literature on social norms; we conclude by describing each pitfall in greater depth.

## Background

Theoretical and empirical literature on social norms exists in sociology, anthropology, social and moral psychology, economics, law, political science, and health sciences. Definitions across these disciplines vary and sometimes contradict each other. The full range of these definitions includes a constellation of social rules ranging from mere etiquette to the most fundamental moral duties [13, 14, 37, 38]. In their simplest definition, social norms are the informal, mostly unwritten, rules that define acceptable, appropriate, and obligatory actions in a given group or society. Current practitioners’ interest in social norms theory mostly draws from the work by Cialdini and colleagues, who defined social norms as one’s beliefs about: 1) what others in one’s group do (descriptive norms); and 2) what they approve and disapprove of (injunctive norms) [39–44]. Influence of norms has been demonstrated empirically. Drawing on Cialdini’s definitions, researchers have demonstrated the influence of social norms on several health-related practices, including: alcohol consumption [45, 46], food intake [47], use of recreational drugs [48, 49], smoking [50], water purification [51], and hand washing [52].

Many theories exist on why people comply with social norms, including when doing so is harmful to self and others. In a recent review, Young [37] identified four main compliance mechanisms: 1) Coordination: people want to achieve a goal that requires coordinated action among group members; to that purpose, they follow what they believe to be common rules for that action; 2) Social pressure: people anticipate social rewards or social punishment for their compliance and non-compliance with a norm; trying to achieve the former and avoid the latter, they follow social norms even when they may prefer not to; 3) Signalling and Symbolism: people want to signal their membership in a given group to self and/or others; to do so, they follow what they think to be the rules specific to that group; 4) Benchmark and Reference points: people internalise rules of what action is considered normal in a given situation, to the point that they follow those rules automatically — what Morris and colleagues called social autopilot in [53].

Social norms are not written in stone; they naturally evolve through time, and sometimes can change very quickly [13, 54–58]. The literature on what works to spark sustainable social norms change in LMIC is still emergent, but growing [16]. In their recent review, Miller and Prentice [15] identified three recurring approaches to change social norms. The first is *social norms marketing*: this strategy was used in the 80s to address college students’ drinking. To change group behaviour, social norms marketing campaigns aim to correct people’s misperceptions of what others in their group do and approve of. In college drinking interventions, for instance, social marketing strategies sent messages like: “85% of students in this university only drink one beer on Saturday and approve of those who do the same”. The second strategy is *personalized normative feedback*, where people receive information on how they are performing against others around them. This strategy, which exploits the influence of (descriptive) norms, has been used, for instance, to reduce energy consumption. By telling people whether they were doing better or worse at saving energy than their neighbours, the intervention achieved on average a considerable reduction in energy consumption [59, 60]. The third strategy is facilitator-led *group conversations*, where participants look critically at existing norms and practices within their group and renegotiate those norms among themselves. A few models exist of how group reflection processes can help achieve change in harmful social norms [18, 20, 61–64]. Studying effective facilitator-led programmes, Cislaghi [65, 66] identified three steps for social norms change: 1) motivation (where participants learn about the detrimental consequences for themselves and others of their compliance with the harmful norm); 2) deliberation (where participants create a new positive norm within their reference group and identify strategies to motivate others in their surroundings); and 3) action (where participants publicly enact their strategies and motivate others to join the group, eventually reaching the critical mass needed for normative change). This final step is often formalized through a public commitment to change.

The current literature, however, does not adequately elucidate the challenges and potential pitfalls of designing change strategies using social norms theory. Here, we list the most critical factors to be taken into account when designing such interventions.

## Eight pitfalls of social norms interventions

As we mentioned, changing health-related social norms is critical to facilitate improvement in people’s relations and wellbeing [67–73]. Effective health promotion programmes should not only help people resist existing harmful expectations, they should also facilitate change in the expectations around them [70, 74, 75]. We have identified the following eight critically-important pitfalls of social norms interventions that can help design such interventions.

### Pitfall #1: Conflating social norms and personal attitudes

**Fig. 1:**
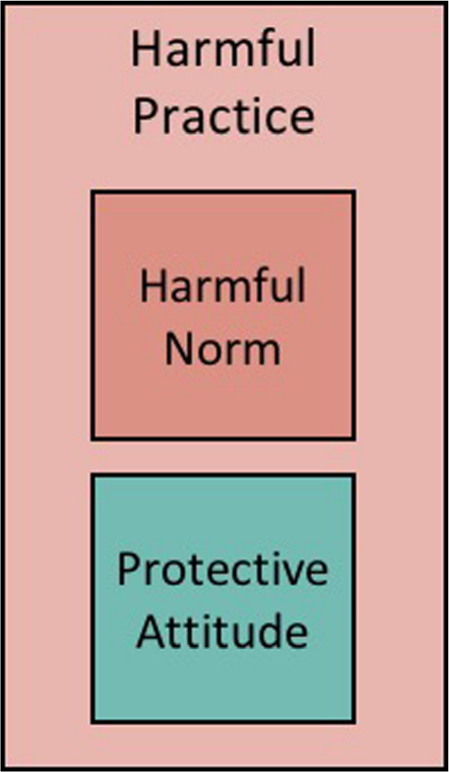
A harmful norm can trump one’s protective attitude, resulting in compliance with a harmful practice

The two psychological constructs—social norms and attitudes—are connected but distinct (social norms can influence attitudes and vice versa). One of the most frequently cited social norms theories, Fishbein and Ajzen’s theory of reasoned action, describes attitudes as internally-motivated judgements that people make about something, such as: “I don’t like going to church” [76]. Social norms, instead, are beliefs about what other people do and approve of, for instance, “People around me go to church and people important to me expect me to do likewise”. The difference is important: one person might attend church not because she or he really wants to (attitude), but to meet the expectations of others (see Fig. 1).

The misalignment between attitude and norm can influence the actions of multiple people in a group, to the point that everyone in the group might hold a protective personal attitude (“I believe that girls should be at least 18 before they marry” ) but think that everyone else holds a different position (“the people around me marry their daughters as soon as they reach puberty, and expect me to do likewise”). This phenomenon is commonly referred to as *pluralistic ignorance* [77, 78]. When most people in a group hold contrasting attitudes and norms, an intervention might achieve change by unveiling the misperception that keeps people bound to the harmful norm. That is, by showing that most people in the group hold the same personal attitudes, interventions might contribute to dismantling the harmful norm.

The difference between attitudes and norms also has implications for social norms measurement. Practitioners who implement an intervention to change social norms should pay attention to the difference between norms and attitudes as they design their measurement strategies, and select some of the tools created specifically to measure social norms “see, for instance: [20, 21]”. Sometimes, however, measures of norms are not available to researchers using existing datasets. Most multi-country datasets (DHS, World Value Survey, MICS, for instance) do not include specific measures of social norms, but they do include measures of personal attitudes (for instance, the DHS includes measures of personal attitudes towards acceptability of violence). Researchers interrogating those datasets often resort to aggregating attitude data at the cluster level, as a proxy for social norms [13, 79, 80]. Note that some researchers have referred to and defined measures of cluster-level attitudes as “collective attitudinal norms” [81, 82].

### Pitfall #2: Focussing exclusively on discordant norms and attitudes

**Fig. 2:**
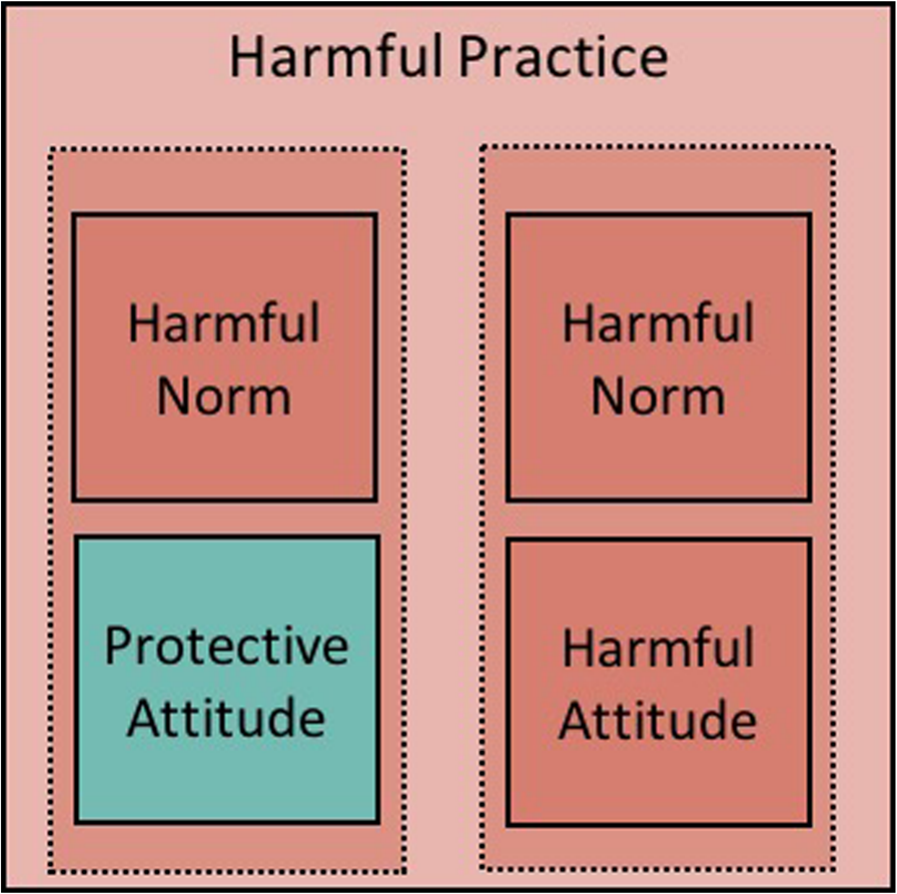
Norms and attitudes can be contrasting or aligned

A tendency exists in the social norms literature, particularly in social psychology, economics, and implementation science, to focus largely on discordance between attitudes and norms (as depicted in Fig. 1). Since the early work on norms and students’ use of alcohol, a large number of empirical studies investigated how discordant norms and attitudes influence people’s practices “e.g. [28, 29, 83–92]”. Norms and attitudes, however, can be aligned: not only can people believe that compliance with a harmful practice is expected of them, they can also have a positive personal attitude towards that practice. Take the example of female genital cutting, for instance. In some places, people might think that “cutting their daughter” is both what’s expected of them and a good thing to do independently of what others do (Fig. 2).

Uncovering the relation between people’s attitudes and norms is critical to intervention design. While correcting misperceptions (as in the campus drinking example) might be an appropriate strategy when norms and attitudes are discordant, it will not serve when people’s attitudes align with the norm. In this latter case, practitioners may first need to change the attitudes of a core group of individuals, and then help them become local change agents, reaching out to the larger group. This might be done, for instance, by providing the group with information on the harmful consequences of a given practice and inviting them to reflect critically on the reasons for the practice. Next, practitioners could help participants devise strategies to motivate others in their settings to join their movement for change (a process that has been referred to as “organised diffusion”) ([30], Cislaghi B, Deeny EK, Cissé M, Gueye P, Shresta B, Shresta P N, Ferguson G, Hughes C, Clark C J: Changing social norms: the importance of “organized diffusion” for scaling up community health promotion interventions, submitted). As the change process starts and the new healthful (or protective) norm spreads within the group, some people’s personal attitudes might not change, but the new norm might induce them to adopt a healthier practice.

In sum, even though there is no universal relationship between attitudes and norms (either may change first), the two nonetheless influence each other in ways that practitioners should study and take account of in their work.

### Pitfall #3: Overlooking protective norms

**Fig. 3:**
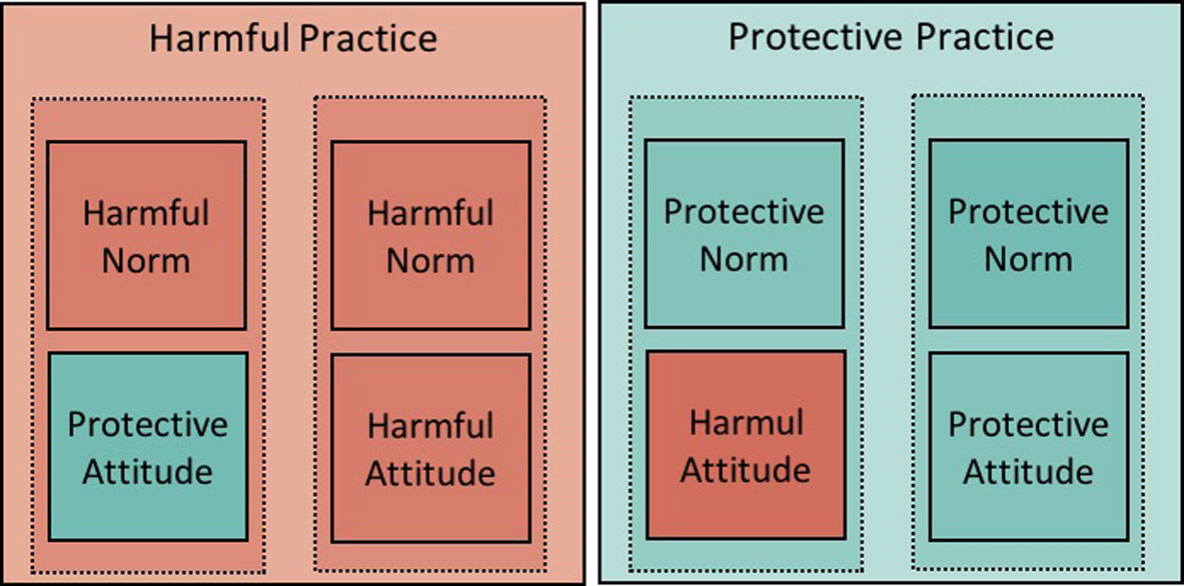
Possible effect of attitudes and norms on the practice (assuming the norm trumps the attitude)

Another implicit bias in development is to see “culture” only as a source of problems rather than as a space for possible solutions. But, in any given cultural context both potentially harmful and potentially protective norms likely exist (See Fig. 3).

As an example, imagine a setting where a norm exists among adolescents under which drinking alcohol is considered a sign of weakness – some readers might have observed such a norm in place in some Scandinavian countries, as well in some of the countries with a quasi-totality of Muslim population (one of the two authors observed this norm to be in place in several areas of Senegal, West Africa). One adolescent might be curious to try alcohol, but fearing community shame might refrain from doing so. As practitioners design their interventions, they would benefit from understanding the protective and harmful roles of existing social norms. Effective interventions might work with local populations in devising strategies to strengthen protective norms, building on existing cultural values and worldviews [93, 94].

### Pitfall #4: Assuming social norms are the sole driver of harmful practices

It is rare (although not completely impossible) for social norms to be the exclusive reason motivating people to engage in a harmful action or practice. As many have observed [95–100], the ecology of factors contributing to a given practice goes well beyond one specific driver. Understanding how norms intersect with other factors is essential to uncover the pathways that motivate people to compliance with harmful practices. Think, for instance, about the work by Bersamin and colleagues [101], who studied what explains young female students’ lack of access to health services. They did find that norms against using the services could be a possible barrier to access, but they also found that focussing on norms alone wouldn’t be adequate: the services themselves must exist; they must be accessible; and women need to know what services are offered and when they can access them.

In addition to understanding the range of factors that influence a given practice, it is important to understand how they interact. For instance, studying how material and social factors affect people’s electricity consumption, Pellerano and colleagues found that extrinsic financial incentives (a material factor) can sometimes reduce the effect of a normative message (a social factor). Their findings suggest that when people feel that they are complying with a new practice for money, they might be less motivated to do so than when they feel they are complying for a “greater” social purpose [102].

**Fig. 4:**
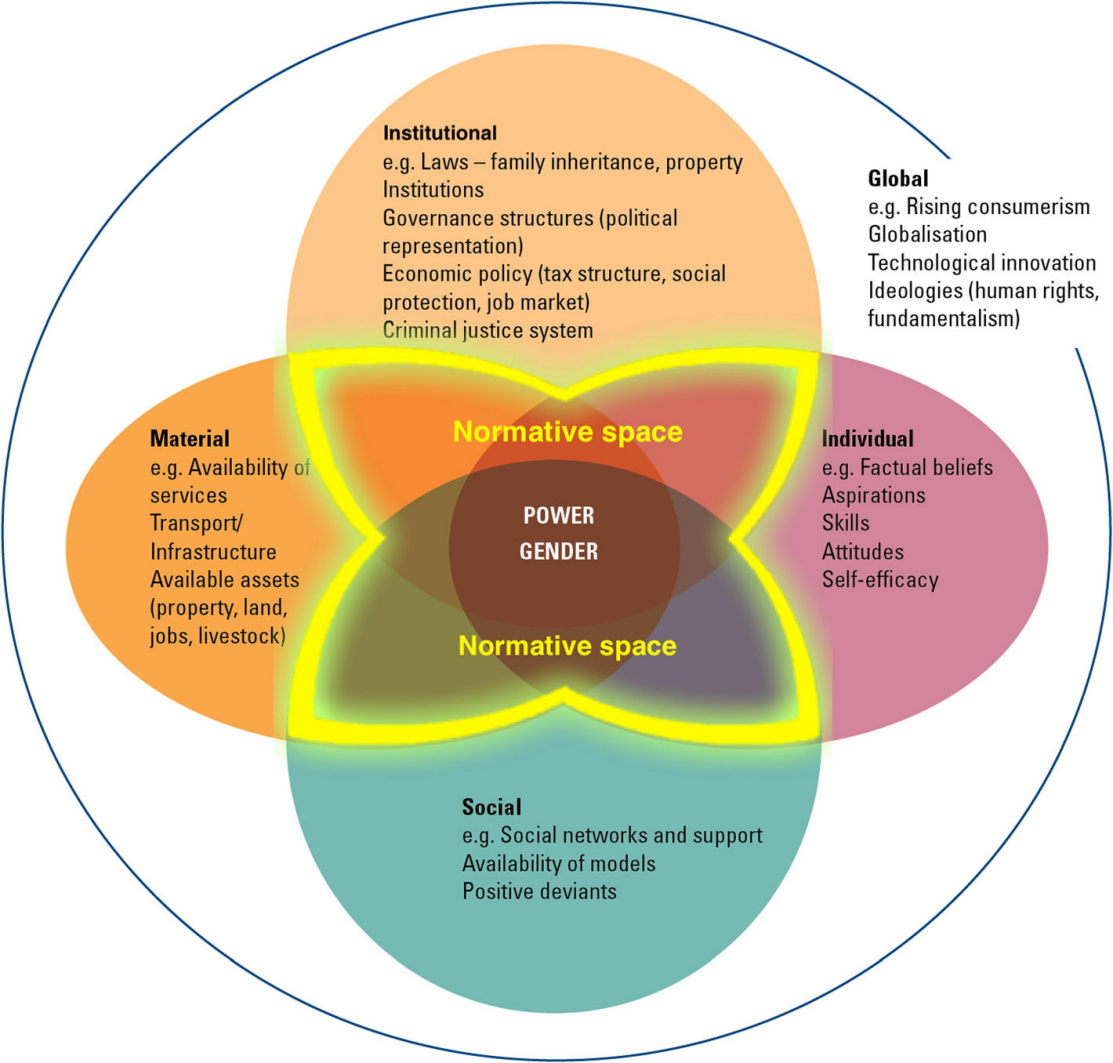
Effective interventions uncover and address the interactions between norms and other factors sustaining harmful practices [Source: 9]

Recently, Cislaghi and Heise [9] offered a practical framework practitioners can use to look at that the ecology of factors contributing to sustaining any given practice. Their framework, which evolves from the well-known ecological framework, includes four domains of influence: institutional, individual, social, and material (see Fig. 4).

Understanding how institutional, individual, social and material factors interact to influence people’s harmful practices can help practitioners design effective interventions that include a social norms perspective. Similar coordinated change could be achieved by organisations collaborating both amongst themselves and with the population whose actions are influenced by these factors.

### Pitfall #5: Confusing the prevalence of a social norm with its influence

Partly due to practitioners’ increased efforts to measure social norms as part of programmatic monitoring and evaluation, data on the prevalence of specific norms is starting to appear at conferences, and in reports and academic papers. These data are often presented to explain the extent to which a given norm sustains a particular practice. However, most studies thus far have invested more time, thought, and resources in measuring the *prevalence* of a norm (i.e. how many people in a specific group hold normative belief X), than measuring its *influence* (i.e. how many people do X because of the social norm). Scholars of social norms have advanced several hypotheses of what might determine the strength of a norm [14, 19]. Cislaghi and Heise [19], for instance, suggested that characteristics of a practice can affect the influence a norm might exert. One of these characteristics is “detectability”: if a norm exists that “you should do X”, but X is completely undetectable (that is: nobody can know whether others are doing X or not), the norm might have less influence on people’s compliance with X than in the opposite case, when compliance is very detectable.

That is not to say that the relation between a norm X and a related undetectable practice can’t have harmful effects nonetheless. In similar cases, people might never disclose their non-compliance with X, but their non-disclosure might result in harm to self or others. Think of a setting where a social norm exists that says, “you shouldn’t have sex before marriage”. Some adolescents may nevertheless have sex before marriage despite the norm. These same adolescents might not want to disclose to others their sexual activity, possibly anticipating social punishment for it. Their non-disclosure might then limit their capacity to learn about and access modern contraceptive methods (potentially increasing their risk of an unwanted pregnancy or of contracting an STI).

Understanding the influence of one or multiple norms over a given practice should be a priority for effective intervention design. This could be done through qualitative research (see below), possibly coupled with quantitative measures exploring associations between prevalence of normative beliefs and prevalence of the practice of interest, at the cluster level.

### Pitfall #6: Neglecting the indirect influence of social norms

**Fig. 5:**
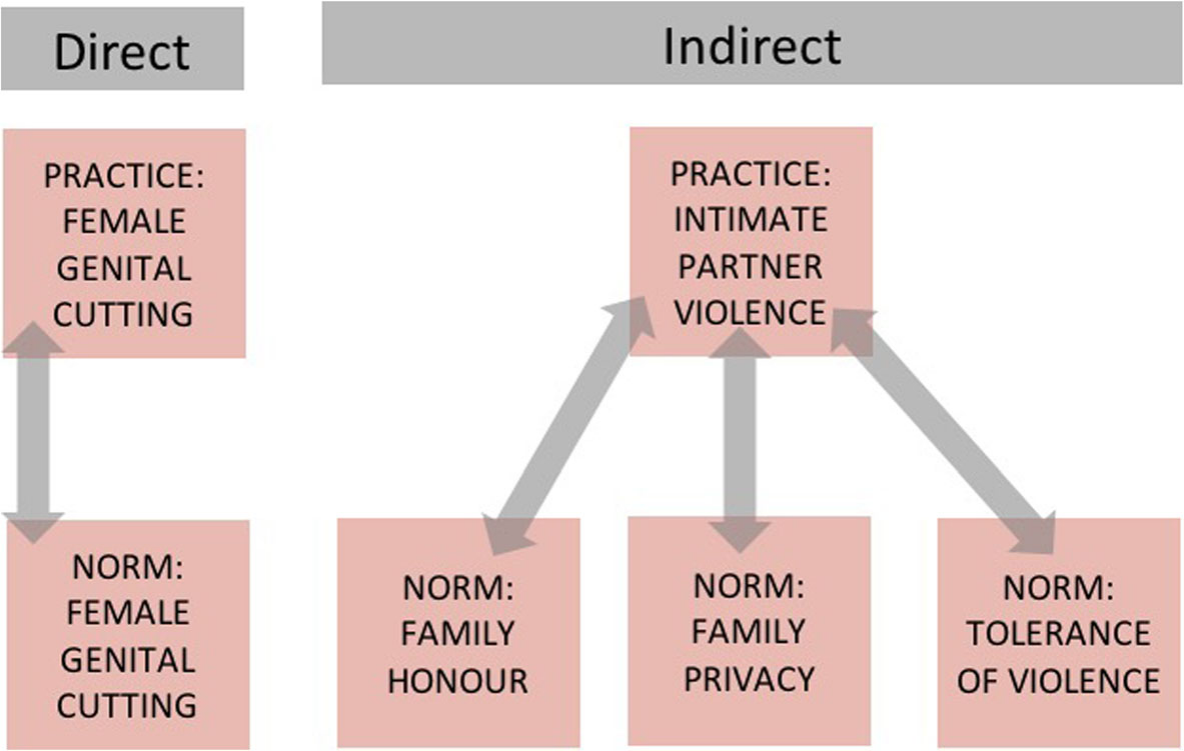
Two possible relations (direct and indirect) between a harmful practice and social norms

Practitioners studying the effect of social norms on a practice X (such as child marriage) might be tempted to look for a norm that people are expected to do X (marry their daughter young). An example comes from female genital cutting, where research conducted in West Africa demonstrated that, in some areas, the practice “cutting your daughter” was sustained by the norm “people around here think that only girls who are cut are respectable” [30, 103, 104]. We call situations where the norm and the behaviour are matched, a *direct* relation between the practice and the norm [19]. But a practice X can also be *indirectly* sustained by multiple norms. Intimate partner violence (IPV), for instance, might be sustained by the norms: “you’re not supposed to intervene in another family’s affairs”; “women are not supposed to disclose family matters to others”; and “women are supposed to keep the family together at any cost” (See Fig. 5).

Intervention design should thus be informed by exploratory formative research, specifically tailored to develop an in-depth understanding of the relations between various constellations of norms, and their direct and/or indirect effects over the practices of interest. Qualitative strategies and techniques for diagnosing social norms are available elsewhere [20, 21]. Vignettes are often recommended as a good method to diagnose social norms e.g. [13, 105]. Vignettes are short stories that simulate contextual elements of a practice under study (for instance, in the case of child marriage, they might include a father telling his friends that he’s found a husband for his 12-year old daughter). Vignettes are usually followed by a series of questions to probe aspects of the respondent’s beliefs.

Note, however, that vignettes present participants with a set of specific scenarios, relational dynamics, and social contexts in which the action of interest is carried out; in other words, vignettes are selective in the contextual elements they simulate [106]. Their selective nature can be both an advantage and a disadvantage. Since they are selective, vignettes don’t easily allow participants to deviate from the scenario that researchers present to them; researchers have already made choices about who influences who (“the reference group”), the place where the action of interest happens, and the direct or indirect norm that sustains the practice. They are better used when researchers already have an idea of what norms sustain the practice of interest in a particular context. Open-ended techniques are often better suited to situations where little is known about the norms that sustain a given practice. These methods might include participatory approaches that invite participants to discuss all possible contextual elements of the practice of interest. Themes presented to participants might include: in what ways is the action of interest carried out in their context? Who carries it out, who doesn’t and what explains this difference? Where is the action carried out? Who witnesses it? Who would approve or disapprove of it? Vignettes might follow as a strategy to uncover the influence of social norms in a specific number of selected scenarios, built from the previous description of the context in which the action takes place.

### Pitfall #7: Publicising the wide prevalence of a harmful social norm

Social norms theory can help recognise the risks in designing campaigns that highlight the great number of people complying with a harmful practice. Those concerned about an issue frequently attempt to motivate change by publicizing the size of the problem: “1 in 3 women globally are abused by their partner”; or: “The average American intakes 44.7 gallons of sugary soda each year”. Because descriptive norms (beliefs about what others do) can influence people’s behaviour, such campaigns can unwittingly reinforce a practice [15, 107, 108]. Even though this point is quite well established in the theoretical and empirical literature, there are still questions about how this finding can inform effective interventions. In designing intervention strategies, caution and serious thought should be given to whether it will be beneficial to use messages that raise awareness in the general population of the size of a problem, like: “65,000 12-year-old girls were married this year in this region alone”. We don’t yet know who is most likely to be influenced by such messages. It might be that these messages sway those who already hold personal attitudes in favour of the harmful practice; but a concrete risk exists that similar messages might backfire, pushing some previous non-compliers to comply with the harmful norm [109].

### Pitfall #8: Engineering social norms change from the outside-in

Local worldviews, norms and attitudes intertwine to sustain cultural practices in ways that may be difficult for practitioners to fully decipher in culturally unfamiliar contexts. It can thus be dangerous to design a new desired system of norms from the “outside”. The consequences of the new normative equilibrium might be as harmful as the practices it is meant to replace. Practitioners should thus strive to design people-led interventions that help participants develop both internal motivations to change local norms and strategies to do so in ways that are compatible with the local cultural and social context [65, 110, 111].

Likewise, social norms systems can be highly self-protective. Because those who challenge the norm might face social punishment, their failed attempts to challenge the equilibrium might result in greater harm for them than compliance. Others witnessing this backlash might be discouraged to join future movements for change. Asking people instead to plan and lead the movement for change builds their capacity to identify key change actors, join with them, and then move to action when they feel they have achieved the collaboration of other key people in their network. For the same reason, it might be ineffective (if not dangerous) to spread intervention efforts across geographical or social clusters. Concentrated interventions that work with people’s entire social networks might be both more effective and less likely to elicit backlash against those first agents of change who venture to unsettle the normative equilibrium.

## Conclusion

This paper offers some practical reflections for those designing interventions addressing social norms. At this particular moment of research and practice, eight pitfalls seem to be particularly critical for achieving effective normative change. The corresponding learnings for practitioners are: 1) Social norms and attitudes are different; 2) Social norms and attitudes can coincide; 3) Protective norms can offer important avenues for effective social change; 4) Harmful practices are sustained by a matrix of interacting factors; 5) The prevalence of a norm is not necessarily a sign of its strength; 6) Social norms can exert both direct and indirect influence; 7) Publicising the prevalence of a harmful practice can recruit more people to the practice; 8) People-led social norm change is both the right and the smart thing to do. As the understanding of how norms evolve in LMIC advances, practitioners will develop greater understanding of what works to help people lead change in harmful norms within their contexts. Awareness of these pitfalls has helped several programmes in the past increase the effectiveness of their interventions. Hopefully, others will benefit from these reflections as well.
